# Metabolic Syndrome and Risk of Cervical Human Papillomavirus Incident and Persistent Infection

**DOI:** 10.1097/MD.0000000000002905

**Published:** 2016-03-07

**Authors:** Xin Huang, Qun Zhao, Pingting Yang, Ying Li, Hong Yuan, Liuxin Wu, Zhiheng Chen

**Affiliations:** From the Department of Preventive Medicine, School of Medicine, Hunan Normal University (XH); Department of Health Management (XH, QZ, PY, LW, ZC); Department of Health Management (XH, QZ, PY, LW, ZC); Department of Clinical Pharmacology Center (YL, YH); the Third Xiangya Hospital, Central South University, Changsha, Hunan; and Institute of Aviation Medicine (LW), Beijing, China.

## Abstract

Few studies have been conducted on the relationship between metabolic syndrome (MetS) and cervical human papillomavirus (HPV) incidence and persistent infection.

We performed a prospective cohort study including 8598 female employees in Hunan, China. First, the subjects were stratified into HPV-negative (N = 7282) and HPV-positive (N = 1316) subgroups, according to the results of an HPV DNA test at baseline. Second, comparisons of the risks of HPV incident and persistent infection between MetS-positive (exposed) and MetS-negative (unexposed) groups were conducted among the HPV-negative and -positive subgroups, respectively.

There were 976 (11.39%) subjects diagnosed with MetS and 1316 subjects diagnosed with HPV infection at baseline. The 12-month cumulative incidence of any type of HPV and high-risk type HPV were 7.28% (530/7282) and 6.26% (456/7282), respectively. Obesity was a modifier of the association between MetS and HPV incident infection. As long as obesity presented, MetS and hypertriglyceridemia were significantly associated with an increased risk of HPV incident infection (any-type or high-risk type) (adjusted risk ratios (RR) were 2.88 (95% confidence interval (CI): 1.16, 7.19) and 3.29 (95% CI: 1.47, 7.38), respectively). Among those infected with HPV at baseline, the 12-month type-specific persistence rates were 51.67% and 53.38% for any-type and high-risk type HPV, respectively. No interaction was found between obesity and MetS with regard to the risk of HPV persistence. After adjustment for confounding factors, MetS was still associated with increased risk of any-type HPV persistence (RR_adj_ = 1.21, 95% CI: 1.05, 1.41) and high-risk type HPV persistence (RR_adj_ = 1.25, 95% CI: 1.09, 1.46). No single metabolic component was associated with the risk of HPV persistence.

The prevalence of MetS was 11.39% among the Hunan female occupational population. MetS was associated with an increased risk of persistent cervical HPV infection and also with an increased risk of HPV incident infection when obesity presented as well.

## Abstract 2

Supplemental Digital Content is available in the text

## INTRODUCTION

With an estimated 485,300 new cases and 235,730 deaths in 2013, cervical cancer ranks as the 4th most common cancer among women in worldwide.^1^ Detection of the same high-risk (HR) type human papillomavirus (HPV) infection for a certain time period (HPV persistence) is considered to be a prerequisite for cervical cancer development.^2^ Although efforts have been made, the mechanisms that lead to persistent infection with HPV remain largely unknown.^3^ The underlying risk factors may include age, tobacco use, multiple sexual partners, low socioeconomic status, and long-term use of oral contraceptives.^4–6^

Metabolic syndrome (MetS) is defined as having 3 or more of the following components^7^: central obesity, hyperglycemia, hypertriglyceridemia, hypertension, and low HDL. Growing epidemiological evidence indicates that the presence of MetS would not only increase the risk of cardiovascular disease^8^ but also increase the risk of several different cancer diseases, such as colorectal cancer, breast cancer, lung cancer, prostate cancer, and bladder cancer.^9^ The latest researches suggest that MetS or MetS components are also risk factors for cervical cancer.^10–13^ One or more of the following factors may contribute to the increased risk of cervical cancer among MetS patients: increased risk of HPV incident infection; decreased possibility of HPV clearance among the infected (increased risk of HPV persistence); and altered prognosis of those with persistent HPV infection. However, it is unclear how many of these factors contribute to the increased risk of cervical cancer and exactly how they may contribute. To answer those questions, the relationship between MetS and HPV infection should be studied. To the best of our knowledge, only 2 previous studies have discussed the associations and reached inconsistent conclusions. Liu et al^14^ claimed that obesity status was a modifier between HPV infection and MetS: decreased HPV prevalence was only found among obese women with MetS but not among nonobese women with MetS. In contrast, Kuo and Fujise^15^ found no association between MetS and HPV infection. Moreover, a lack of temporality was inherent in those cross-sectional studies, and thus the associations between MetS and HPV incident and persistent infection have not been addressed. Therefore, the above-mentioned questions cannot be answered based on the existing published data. Given the inconsistent relationship between MetS and HPV infection and the paucity of prospective studies on the associations between MetS and HPV incident and persistent infection, we performed a prospective cohort study in Hunan, China to estimate the associations between MetS and HPV incident and persistent infection, and to test whether those associations were modified by obesity status.

## METHODS

### Study Population

This cohort study was performed at the Third Xiangya Hospital of Central South University (TXYHCSU). All of the female employees (N = 8992) from 373 organizations were enrolled in our study when they attended the annual employee health exam at the TXYHCSU between January 2010 and June 2014. The selected organizations were administrative institutions or local government units, arranging health exams for employees (including the retired) every year. All of the participants were followed for 12 months following enrollment. The subjects (N = 173) who were maiden, pregnant, or planned to become pregnant in the next year; were taking immunosuppressants; or had undergone amputation of the cervix at baseline were excluded. Furthermore, women (N = 194) who may already have had persistent HPV infection at enrollment (with cervical thin prep cytologic test results worse than atypical squamous cells of undetermined significance at baseline) were also excluded. Another 27 subjects were lost to follow-up and were excluded from analysis because of death or job change, resulting in a final sample size of 8598.

### Data Collection

All of the study procedures were approved by the human investigation committees at the TXYHCSU and Central South University. The eligible subjects were informed about the cohort study upon their arrival at the hospital for their annual health exam. After obtaining written consent, an in-person interview was conducted at baseline by trained interviewers using a standardized and structured questionnaire. The questionnaire covered information on demographics, reproductive history, lifestyle factors, medical history including a diagnosis of hypertension or diabetes, and prescription drug use for hypertension, diabetes, or lipid-lowering.

Cervical specimens were collected both at the time of enrollment and 12 months later by qualified gynecologists with cervical brushes. The brush heads with collected specimens were stored in a 4 °C fridge in capped bottles with preserving liquid and processed for HPV DNA genotyping within 1 week. HPV genotyping by HybriMax was performed with an HPV GenoArray Test Kit (HybriBio Ltd., Chaozhou, China) according to the manufacturer's instructions.^16^ Briefly, after the steps of DNA extraction from cervical cells, gene amplification (PCR), and diversion hybridization, 21 types of HPV could be detected, including 15 high oncogenic risk types (HR-HPV: 16, 18, 31, 33, 35, 39, 45, 51, 52, 53, 56, 58, 59, 66, and 68) and 6 low oncogenic risk types (low-risk type HPV LR-HPV: 6, 11, 42, 43, 44, and 81).

Other physical examinations were conducted with the same methods described in our previous study.^17^ Briefly, the average of 3 seated blood pressure measurements after 15 minutes resting was recorded by skilled physicians. Blood samples were collected at 08:00 to 10:00 AM after a fasting period of 12 hours. Concentrations of fasting blood glucose and triglycerides were determined using an enzymatic colorimetric assay. The high-density lipoprotein cholesterol (HDL-c) concentration was measured by lipoprotein electrophoresis.

### Variable Definition

HPV incident infection (HPV (−, +)) was defined as individuals without HPV infection at baseline but who were infected with any types of HPV 12 months later. HR-HPV incident infection (HR-HPV (−, +)) was defined as individuals without any type of HPV infection at baseline but who were infected with high-risk types 12 months later. Type-specific HPV persistence (HPV (+, +)) was defined as positivity for the same HPV DNA type in cervical samples collected at baseline and 12 months later from the same woman. If a woman was found to be infected with HPV at baseline, but the second HPV test 12 months later showed a negative result or a different HPV type on 2 occasions, then she was considered to have cleared that HPV type (HPV (+, −)). Type-specific HR-HPV persistence (HR-HPV (+, +)) was defined as persistent HPV infection with high-risk HPV types. According to the National Cholesterol Education Program Adult Treatment Panel III (NECP-ATP-III) criteria,^7^ MetS was defined as having 3 or more of the following 5 components: central obesity – waist circumference ≥80 cm (for females); elevated blood pressure – systolic blood pressure (SBP) ≥130 mmHg, diastolic blood pressure (DBP) ≥85 mmHg, a previous diagnosis of hypertension, or the current use of anti-hypertension medications; hypertriglyceridemia – triglycerides ≥1.69 mmol/L or taking lipid-lowing medications; low HDL – HDL-c < 1.29 mmol/L (for females); and hyperglycemia – fasting glucose ≥5.6 mmol/L, previous diagnosis of diabetes, or the current use of antidiabetes medications. All of the study subjects were classified into MetS-positive (exposed) and MetS-negative (unexposed) groups according to their metabolic health status. The recommended body mass index (BMI) value for the Asian population was considered to classify obesity (BMI ≥ 27.5 kg/m^2^).

### Statistical Analysis

**FIGURE 1:**
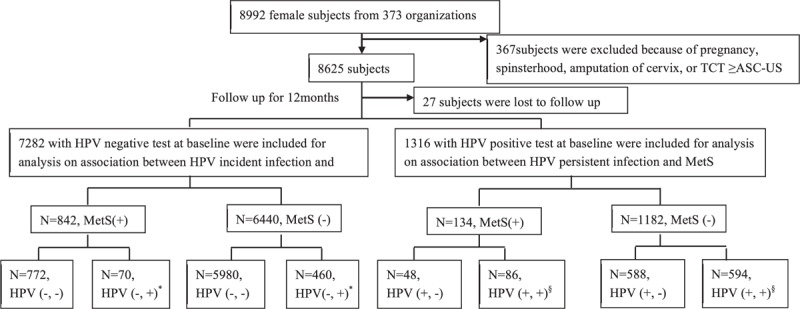
Flow chart of the study design. ^∗^HPV incident infection (HPV (−, +)): with no HPV infection at baseline, but infected with any type of HPV infection 12 months later. ^§^HPV persistent infection (HPV (+, +)): positivity for the same HPV DNA type in cervical samples collected at baseline and 12 months later. ASC-US = atypical squamous cells of undetermined significance, HPV = human papilloma virus, MetS = metabolic syndrome, TCT = thin prepcytologic test.

The flow chart of our analytical scheme is presented in Figure 1. Briefly, we analyzed the data in 2 steps. First, according to the results of an HPV test at baseline, all of the eligible subjects were classified into HPV-negative and -positive subgroups. Second, the data were analyzed separately within each subgroup. The risk of HPV incident infection was compared between the exposed and unexposed groups among the HPV-negative subgroup. Meanwhile, the risk of type-specific persistent HPV infection was compared between the exposed and unexposed groups among the HPV-positive subgroup. Chi-square tests were used to compare the distributions of baseline characteristics between the exposed and unexposed groups. To test whether obesity was a modifier of the association between MetS and HPV infection, a stratified analysis and the Breslow-Day test were adopted. The relative excess risk due to interaction (RERI) and the ratio of risk ratios (RRs) were calculated to assess the effect of modification under the additive and multiplicative models.^18^ If there was homogeneity between the different layers (*P* ≥ 0.05), then obesity status was considered as a confounding factor. Meanwhile, in cases in which there was heterogeneity (*P* < 0.05), the associations were separately described in the obese and nonobese layers. Multivariate log-binomial regression models were used to calculate the RR and 95% confidence intervals (CIs) for the associations between MetS, metabolic components, and the risk of any-type HPV incident infection, HR-HPV incident infection, type-specific persistent infection with any type of HPV, and type-specific persistent HR-HPV infection. Potential confounding variables included in the models were age at baseline (21–29, 30–39, 40–49, and ≥50 years), years of education (≤12, >12 years), marital status (married or other status [including unmarried, divorced, and widowed]), menarche age (<14, ≥14 years), age at first sexual intercourse (<20, ≥20 years), number of sexual partners in the past 6 months at baseline (≤1, >1), and obesity status at baseline (yes or no, if obesity was considered as a confounding factor). When each of the metabolic components was treated as an exposure of interest, the other metabolic components except for the one of interest were additionally included in the models as adjustment variables. Statistical significance was assessed at the 5% level (2-tailed test). All of the analyses were performed using SAS software, version 9.2 (SAS Institute, Inc., Cary, NC).

## RESULTS

Of the 8598 study subjects, 976 (11.35%) were diagnosed with MetS, 2418 (28.12%) with central obesity, 1766 (20.54%) with elevated blood pressure, 1306 (15.19%) with hypertriglyceridemia, 606 (7.05%) with low HDL-c, and 1572 (18.28%) with hyperglycemia. Compared to the unexposed group, women with MetS were more likely to be older (Table 1). There were no significant differences in the distribution of years of education, marital status, menarche age, age at first sexual intercourse, or number of sexual partners in the past 6 months between the exposed and unexposed groups. The prevalence rates of any-type HPV and HR-HPV at baseline among our population were 15.31% and 13.07%, respectively (Table 2). HPV types 52 (4.11%), 58 (2.42%), 81 (2.38%), 16 (1.70%), and 53 (1.59%) had the highest prevalence rates. The distributions of characteristics at baseline among the HPV-positive and -negative subgroups were similar to that among the overall population (Table 1).

**TABLE 1:**
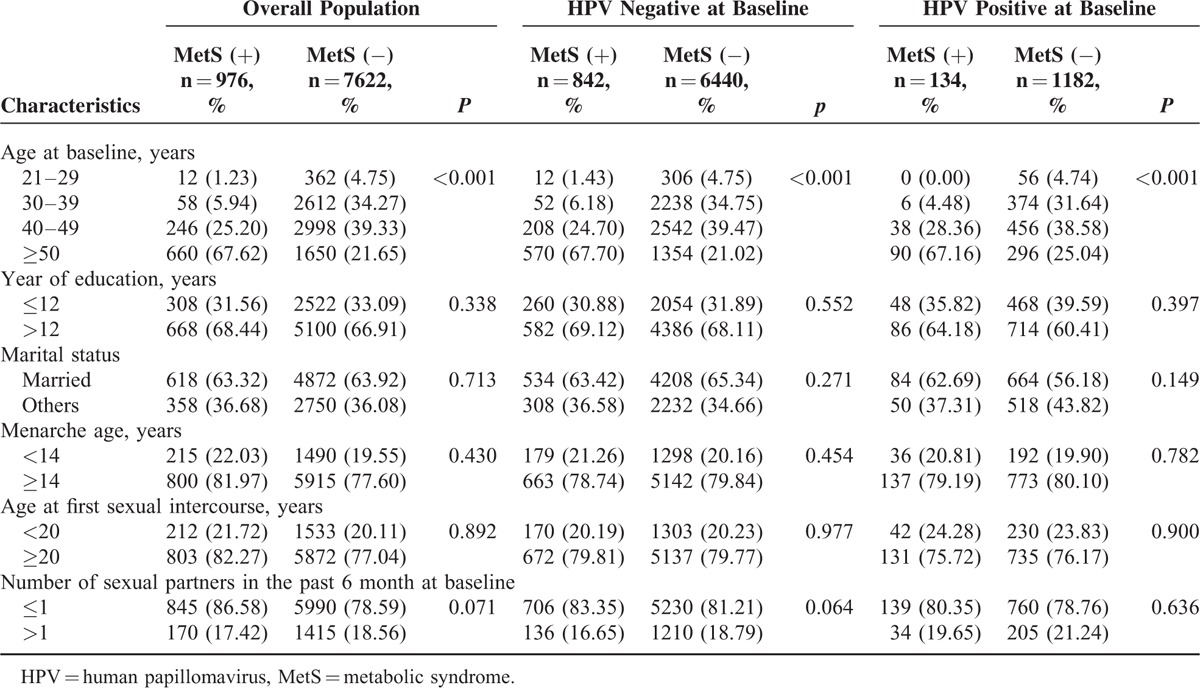
Distributions of Baseline Characteristics Between Metabolic Syndrome Exposed and Unexposed Group

**TABLE 2:**
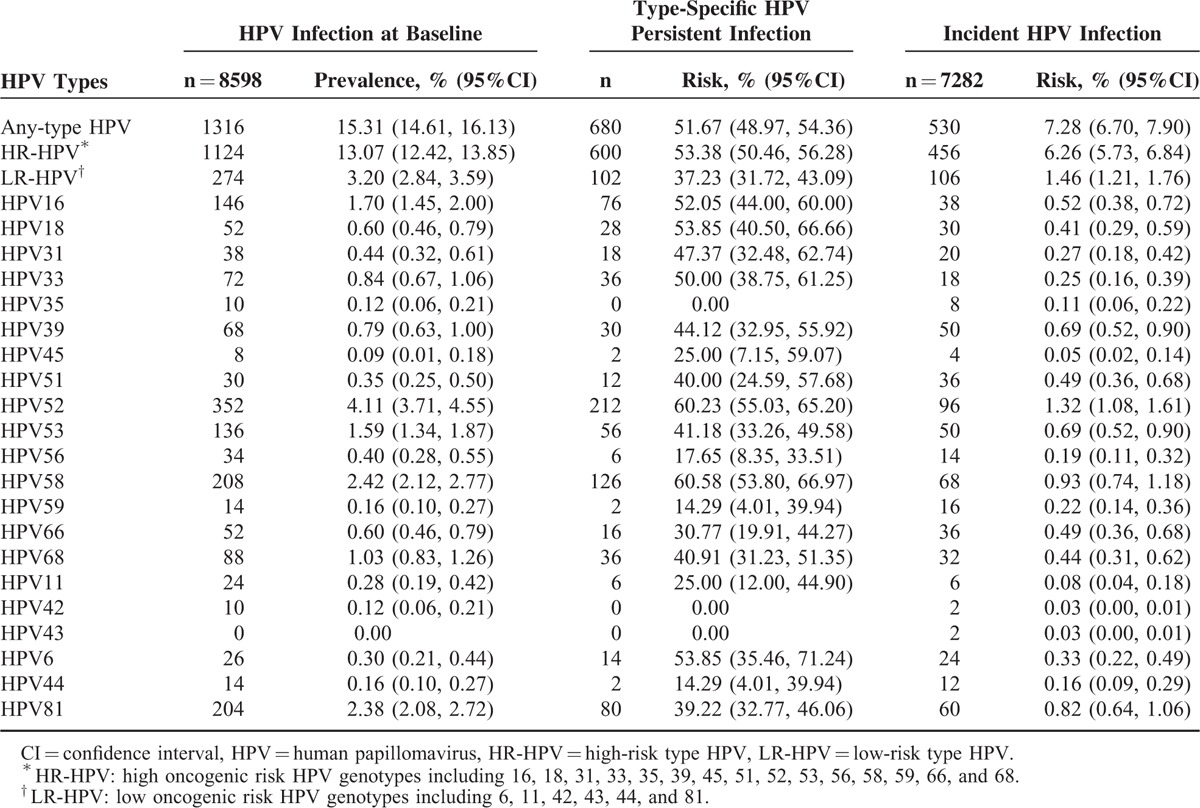
The Distribution of Infected HPV Types Among Study Subjects

The 12-month cumulative incidences of any-type HPV, HR-HPV, LR-HPV, and multiple HPV type infection were 7.28%, 6.26%, 1.46%, and 1.10%, respectively (Table 2). HR-HPV types 52 (1.32%), 58 (0.93%), 53 (0.69%), 39 (0.69%), and 16 (0.52%) had the highest cumulative incidences of HR-HPV. The distribution of HPV genotypes was similar between the different exposure groups (data not shown). The newly acquired HPV types among the obese group all belonged to high oncogenic risk HPV types. Breslow-Day tests showed that obesity was a modifier of the association between MetS and HPV incident infection (Table 3). The interaction was positive on both the multiplicative and additive scales (Supplementary Table 1). Among the nonobese group, MetS or single metabolic components were not associated with HPV incident infection (Table 4). However, among the obese group, even after adjustment for confounding factors, MetS and hypertriglyceridemia were significantly associated with an increased risk of HPV incident infection (any-type or high-risk type) (for MetS, RR_adj_ = 2.88, 95% CI: 1.16, 7.19; for hypertriglyceridemia, RR_adj_ = 3.29, 95% CI: 1.47, 7.38) (Table 5).

**TABLE 3:**
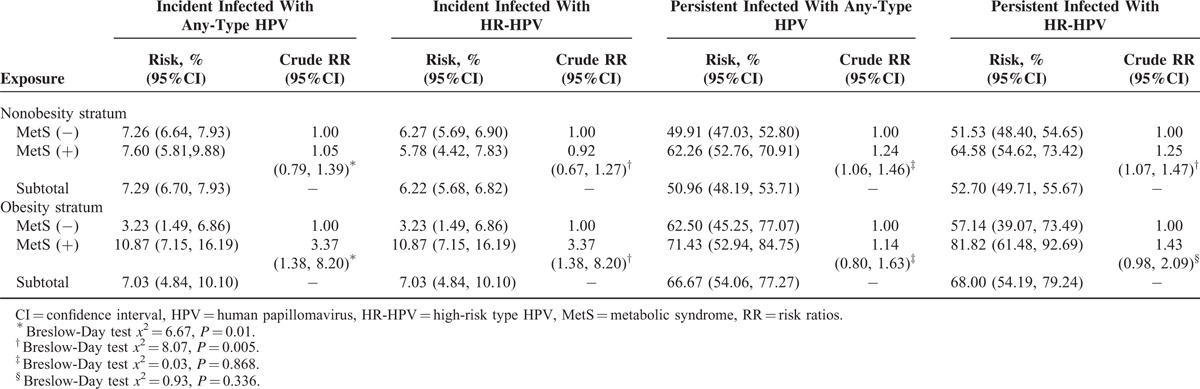
Obesity Status Stratified Analysis on the Association Between Metabolic Syndrome and HPV Incident and Persistent Infection

**TABLE 4:**
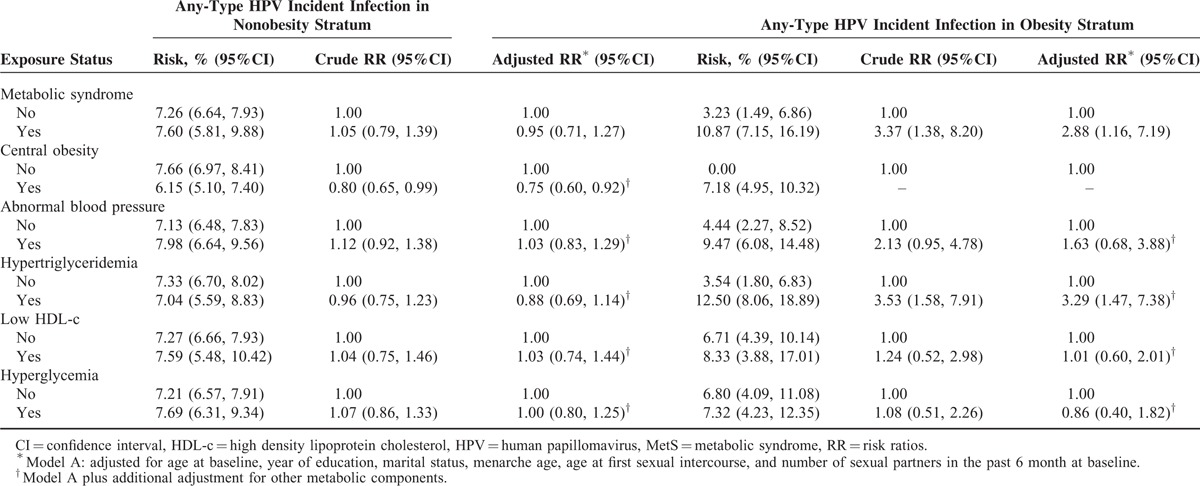
Association Between Metabolic Health Status and Risk of Any Type of Cervical HPV Incident Infection by Specific Obesity Status

**TABLE 5:**
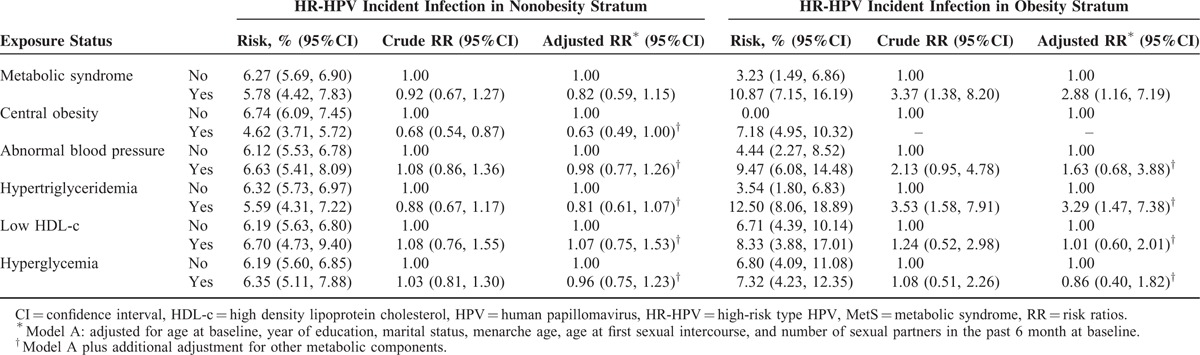
Association Between Metabolic Health Status and Risk of Cervical HR-HPV Incident Infection by Specific Obesity Status

The 12-month type-specific any-type HPV and HR-HPV persistence rates were 51.67% and 53.38%, respectively. HR-HPV types 58 (60.58%), 52 (60.23%), 18 (53.85%), 16 (52.05%), and 33 (50.00%) had the highest type-specific HR-HPV persistence rates. No multiplicative or additive interactions were found between obesity and MetS for the association between MetS and the risk of HPV persistence (Table 3 and Supplementary Table 1). The 12-month type-specific any-type HPV and HR-HPV persistence rates among the MetS-exposed population were 64.18% and 67.80%, respectively, which was higher than that among the unexposed group (the type-specific any-type HPV persistence was 50.25% and the type-specific HR-HPV persistence was 51.69%). After adjustment for confounding factors, MetS remained significantly associated with type-specific any-type HPV persistence (RR_adj_ = 1.21, 95% CI: 1.05, 1.41) and type-specific HR-HPV persistence (RR_adj_ = 1.25, 95% CI: 1.09, 1.46). No single metabolic component was significantly associated with type-specific any-type or high-risk type HPV persistence (Table 6). Additional adjustment for occupation, parity history, smoking, and drinking, the multivariate analysis provided similar results (Supplementary Table 2).

**TABLE 6:**
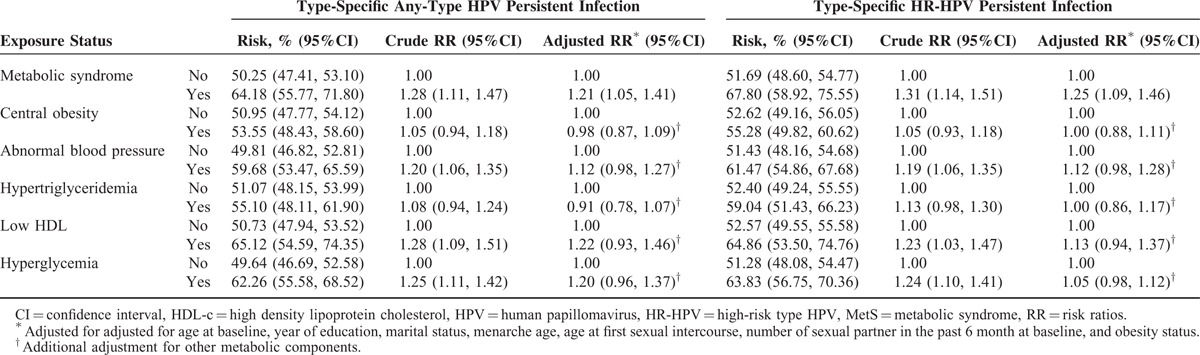
Association Between Metabolic Health Status and Risk of Type-Specific HPV Persistent Infection

## DISSCUSSION

Our study is the first to examine the associations between MetS and HPV incident and persistent infection. Our results support the hypothesis that MetS is associated with an increased risk of persistent cervical HPV infection and also with an increased risk of HPV incident infection when obesity is presented.

Our cohort study showed that the MetS prevalence was 11.39% (95% CI: 10.73, 12.08%) among the female occupational population in Hunan, China. The prevalence of MetS varies in different countries and is approximately 7% to 56% in females worldwide.^19^ The prevalence of MetS in our study was similar to the female professional population in Beijing (10.2%)^20^ and to female adults in Shanghai (12.8%).^21^ However, it was lower than the 17.8% found among female adults in mainland China.^22^ Although the diagnostic criteria for MetS in those previous studies were the same as ours, MetS prevalence increased as age increased.^19^ Therefore, the disparity could partially be a result of the different demographic characteristics of the populations studied. In addition, the health effects of work could also be involved when comparing our prevalence rate with other nonoccupational populations.

The women enrolled in our study were cytological normal, and the prevalence of any type of HPV was 15.31% (95% CI: 14.61, 16.13%), which was similar to the normal cytology population in China (14.80%–16.79%).^23^ The incidence rate among Chinese females has not yet been addressed. Our results showed that the 12-month cumulative incidence of any type of HPV among the occupational population in China is 7.28% (95% CI: 6.70, 7.90%), which is lower than that in other countries in North America^24^ and Europe.^25^ These differences in incidence may be partially attributable to differences in HPV detection methods, the infecting HPV genotypes, and the characteristics of the populations studied. The median HPV clearance time was approximately 6 to 12 months.^26^ A total of 48.33% (95% CI: 45.64, 51.03%) of the subjects with normal cytology in our study had cleared their infections within 12 months, which was similar to previous studies.^26^

To the best of our knowledge, our study is one of the first investigations to prospectively examine the associations between MetS and the risk of cervical HPV incidence and persistent infection. Only 2 retrospective studies performed by Liu et al^14^ and Kuo and Fujise^15^ have discussed the relationship between MetS and HPV infection. By comparing the prevalence of MetS between 1141 women with HPV infection and 1309 without infection, Kuo and Fujise concluded that no significant association exists between MetS and HPV.^15^ Liu et al^14^ enrolled 1987 women with data on MetS and observed no statistically increased risk of HPV infection among nonobese women with MetS. However, a lower risk of HR-HPV infection among obese women with MetS was observed in his study. There are several concerns when comparing our study with those 2 studies. First, the primary infecting HPV genotypes among the Chinese population are quite different from that of Caucasians; the genotypes of HPV may play an important role in the pathogenesis of HPV incident and persistent infection.^25^ Whether the association between MetS and HPV varies from HPV genotype to genotype requires further research. Second, the U.S. Food and Drug Administration (FDA) has approved the use of an HPV vaccine since 2006. Some of the subjects enrolled in Liu's study had received the vaccine,^14^ and the study did not discuss whether the strength of the association between obesity with MetS and HPV prevalence was altered by considering the HPV vaccination history. Although we did not collect information on HPV vaccination, the Chinese FDA has not yet approved the vaccine. Moreover, the costs of HPV vaccination would be very expensive for our subjects, involving not only 3 vaccines but also 3 round-trip travelling fees. The number of subjects receiving the vaccine would therefore be small. Thus, if there was vaccination information bias in our study, the effects of this bias would be subtle and unlikely to fully explain the association identified between MetS and HPV incident infection among the obese group, or the association between MetS and HPV persistence. Third, in our study, the samples used for HPV-DNA tests were collected by health professionals; however, self-collected vaginal swabs were used in the studies by Liu and Kuo. Self-collected samples may still suffer to some extent from under-recognizing HPV infection,^15^ and thus, nondifferential misclassification and underestimating the association may potentially be confounding factors in Kuo's study. Moreover, obese women may have trouble reaching their genital regions because of redundant vaginal wall prolapse.^27^ Thus, the quality of self-collected samples among obese women may require further evaluation, and differential misclassification may be involved in Liu's analysis. Further prospective research is required to confirm the role of metabolic alterations in the natural history of HPV infection. Possible biological mechanisms underlying the differential association among obese women with MetS and nonobese women with MetS may involve aggravated insulin resistance, chronic inflammation, and oxidative stress in obese people with MetS.^28^

Excess adipose tissue, chronic inflammation, insulin resistance, and oxidative stress are the common characteristics of MetS.^28^ Previous studies have reported that elevated estrogen and adipokines produced by adipose tissue and elevated systemic levels of inflammatory cytokines could increase the risk of persistent HPV infection.^29,30^ Consistent with those studies, an increased risk of HPV persistence among MetS was also observed in our study.

Previous studies have observed an increased risk of cervical cancer among women with hypertriglyceridemia.^10,11^ Our prospective study further demonstrates that the increased the risk of HPV incident infection may be one of the pathways for this association. Hyperglycemia or low HDL-c is not associated with HPV prevalence or cervical cancer.^10,12^ A significant association between hyperglycemia, low HDL, and HPV infection was also not observed in our study. Several studies have identified elevated blood pressure among cervical cancer patients.^10,13^ However, we found no statistical association between elevated blood pressure and HPV incident or persistent infection. None of the previously published studies were prospective, and a lack of temporality was inherent in those data. HPV infection is an independent risk factor for cardiovascular diseases such as preeclampsia^31^ and coronary artery disease.^15^ Therefore, reverse causality may be involved in those retrospective studies.

Strengths and limitations should be considered when interpreting our study findings. Our study is an occupational population-based prospective cohort study, which minimizes selection, recall, and reverse causation bias. The data contained detailed demographic and lifestyle information, allowing for simultaneous adjustments for several important potential confounding factors. The diagnosis of MetS in our study was based on medical exams, not self-report, and cervical samples were collected by health professionals, not self-collected, which minimizes potential exposure and disease misclassifications. However, the limitations of our study should also be considered when interpreting the study findings. The detection of the same HPV type in women at both exams may be partially due to recurrent infection with the same HPV type. We were unable to determine whether repeated type-specific HPV positivity represented true persistence or reinfection. The major infecting HPV types among the Chinese population are quite different from that of Caucasians; the types of HPV may play an important role in the pathogenesis of HPV incident and persistent infection.^25^ Hence, the generalizability of our results may be limited to the Chinese population. Additionally, we have followed the subjects for only 12 months, the median duration of HPV persistence among MetS-exposed and nonexposed groups require further long-term investigation.

## CONCLUSION

Our study found that the prevalence rate of MetS was 11.39% among the Hunan female occupational population. MetS was associated with an increased risk of persistent cervical HPV infection and also with an increased risk of HPV incident infection when obesity was presented. These findings have important implications for designing cervical cancer prevention strategies.

## Supplementary Material

Supplemental Digital Content
