# Supplementary material for: Metabolic Syndrome and Risk of Cervical Human Papillomavirus Incident and Persistent Infection
Source: Medicine (Baltimore). 2016 Mar 7;95(9):e2905. doi: 10.1097/MD.0000000000002905 (PMC4782868; doi:10.1097/MD.0000000000002905)
Supplement: Supplemental Digital Content [file medi-95-e2905-s001.doc]

**Supplementary 1**

**Table 1** Interaction between metabolic syndrome and obesity on the risk of HPV incident and persistent infection

| Outcome | | MetS(-) | | MetS(+) | | RR (95% CI) for MetS within strata of obesity | RERI^┼^  (95% CI) | ratio of RR^╫^ (95% CI) |
| --- | --- | --- | --- | --- | --- | --- | --- | --- |
|  |  | N with/without outcome | RR (95%CI) | N with/without outcome | RR (95%CI) |  |  |  |
| Incident infected with any-type HPV | | | |  |  |  |  |  |
|  | Obesity(-) | 454/5800 | 1.00 | 50/608 | 1.05(0.79, 1.39) | 1.05(0.79, 1.39) | 1.01(0.24, 1.77)  *p*=0.010 | 3.22(1.27, 8.18)  *p*=0.014 |
|  | Obesity(+) | 6/180 | 0.44(0.20, 0.98) | 20/164 | 1.50(0.98, 2.29) | 3.37(1.38, 8.20) |  |  |
| Incident infected with HR-HPV | | | |  |  |  |  |  |
|  | Obesity(-) | 392/5862 | 1.00 | 38/620 | 0.92(0.67, 1.27) | 0.92(0.67, 1.27) | 1.29(0.43, 2.17)  *p*=0.004 | 3.66(1.42, 9.42)  *p*=0.007 |
|  | Obesity(+) | 6/180 | 0.51(0.23, 1.14) | 20/164 | 1.73(1.13, 2.65) | 3.37(1.38, 8.20) |  |  |
| Persistent infected with any- type HPV | | |  |  |  |  |  |  |
|  | Obesity(-) | 574/576 | 1.00 | 66/40 | 1.24(1.06, 1.46) | 1.24(1.06, 1.46) | -0.07(-0.58, 0.45) | 0.92(0.62, 1.35) |
|  | Obesity(+) | 20/12 | 1.25(0.95, 1.65) | 20/8 | 1.43(1.12, 1.82) | 1.14(0.80, 1.63) | *p*=0.793 | *p*=0.662 |
| Persistent infected with HR-HPV | | |  |  |  |  |  |  |
|  | Obesity(-) | 504/474 | 1.00 | 62/34 | 1.25(1.07, 1.47) | 1.25(1.07, 1.47) | 0.23(-0.29, 0.74) | 1.11(0.76, 1.72) |
|  | Obesity(+) | 16/12 | 1.11(0.80, 1.54) | 18/4 | 1.59(1.29, 1.95) | 1.43(0.98, 2.09) | *p*=0.387 | *p*=0.523 |

Abbreviations: HPV, human papillomavirus; MetS, metabolic syndrome; RR, risk ratio; CI, confidence intervals; RERI, relative excess risk of interaction.

^┼^ Measurement of effect modification on additive scale, 95%CI was estimated by delta method. ^18^

^╫^ Measurement of effect modification on multiplicative scale.

**Supplementary 2**

**Table 2** Association between metabolic syndrome and risk of cervical HPV incident and persistent infection under different statistical models

| Population | Outcome | Model A ^a^ Adjusted RR (95%CI) | Model B ^a^ Adjusted RR (95%CI) | Model C ^a^ Adjusted RR (95%CI) | Model D ^a^ Adjusted RR (95%CI) | Model E ^a^ Adjusted RR (95%CI) |
| --- | --- | --- | --- | --- | --- | --- |
| Obese | Any-type HPV incident infection | 3.08(1.30, 8.00) | 2.97(1.27, 7.55) | 3.04(1.18, 7.27) | 2.90(1.19, 7.25) | 2.96(1.17, 7.21) |
|  | High risk type HPV incident infection | 3.08(1.30, 8.00) | 2.97(1.27, 7.55) | 3.04(1.18, 7.27) | 2.90(1.19, 7.25) | 2.96(1.17, 7.21) |
| Non-obese | Any-type HPV incident infection | 1.02(0.84, 1.34) | 0.99(0.80, 1.28) | 0.98(0.74, 1.30) | 0.98(0.70, 1.30) | 1.01(0.80, 1.28) |
|  | High risk type HPV incident infection | 0.86(0.70, 1.24) | 0.89(0.61, 1.17) | 0.90(0.62, 1.21) | 0.91(0.62, 1.19) | 0.92(0.60, 1.20) |
| Overall | Any-type HPV persistent infection | 1.25(1.10, 1.45) | 1.22(1.08, 1.40) | 1.20(1.05, 1.33) | 1.21(1.05 1.30) | 1.23(1.08, 1.44) |
|  | High risk type HPV persistent infection | 1.29(1.13, 1.50) | 1.28(1.10, 1.50) | 1.25(1.09, 1.40) | 1.26(1.07, 1.42) | 1.25(1.07, 1.44) |

a Model A: adjusted for age at baseline;

b Model A plus additional adjustment for smoking status;

c Model A plus additional adjustment for drinking status;

d Model A plus additional adjustment for occupation;

e Model A plus additional adjustment for parity history.
